# Supplementary material for: Polyphenol and Tannin Nutraceuticals and Their Metabolites: How the Human Gut Microbiota Influences Their Properties
Source: Biomolecules. 2022 Jun 23;12(7):875. doi: 10.3390/biom12070875 (PMC9312800; doi:10.3390/biom12070875)
Supplement: Supplementary file 1 [file biomolecules-12-00875-s001.zip › biomolecules-1774302-supplementary.pdf]

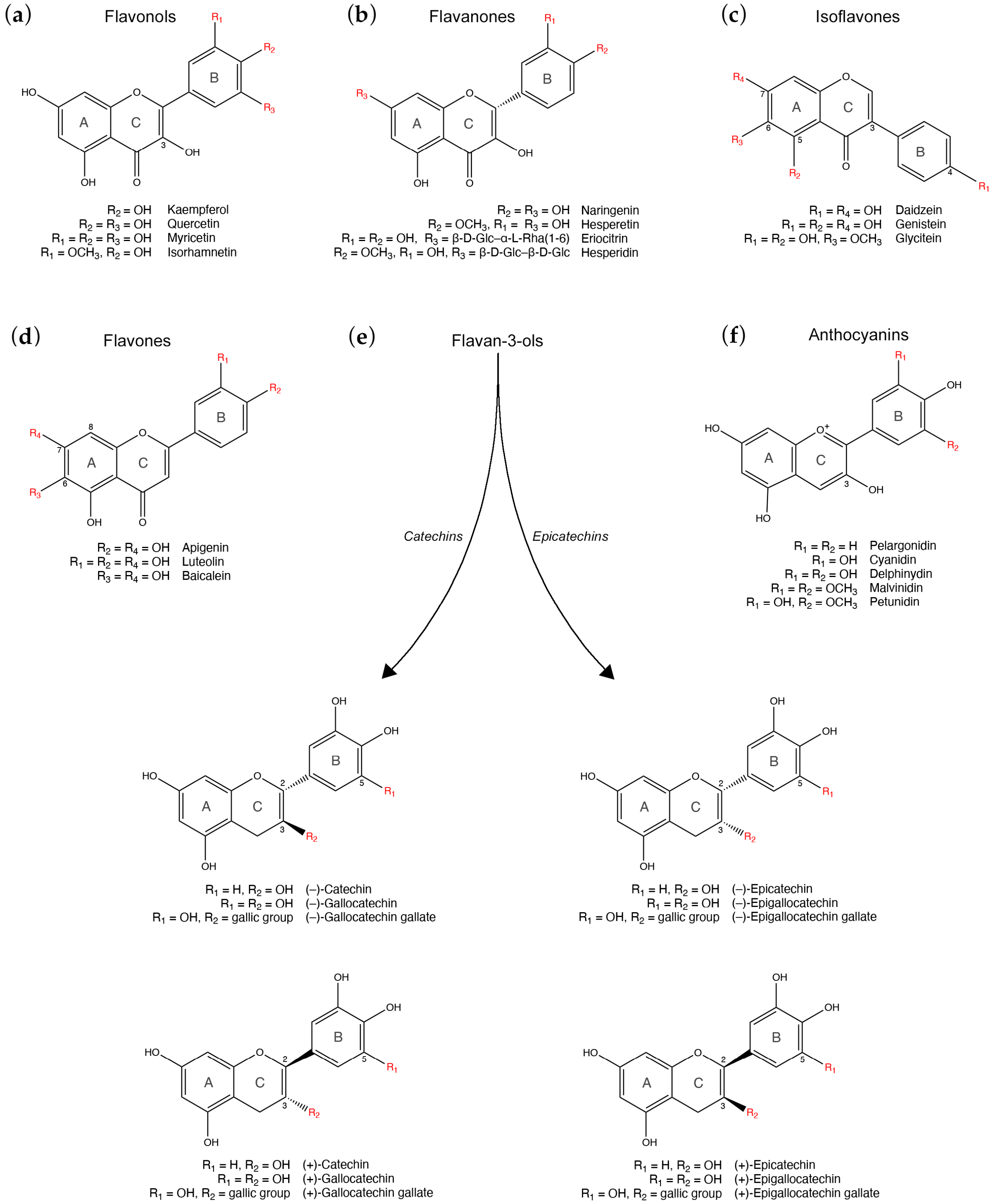

Supplementary Figure S1. Chemical structures of the best-known flavonoid sub-families: (**a**) flavonols; (**b**) flavanones; (**c**) isoflavones; (**d**) flavones; (**e**) flavan-3-ols, divided into catechins and epicatechins according to the orientation of the two chirality centers in position 2 and 3 of the C-ring; and (**f**) anthocyanins. "R" groups point to the position of the main substituents of the 3-hydroxyflavone backbone, whose diversity is responsible for the vast heterogeneity of compounds within each sub-family. When no substituent is specified, the presence of a hydrogen is implied.

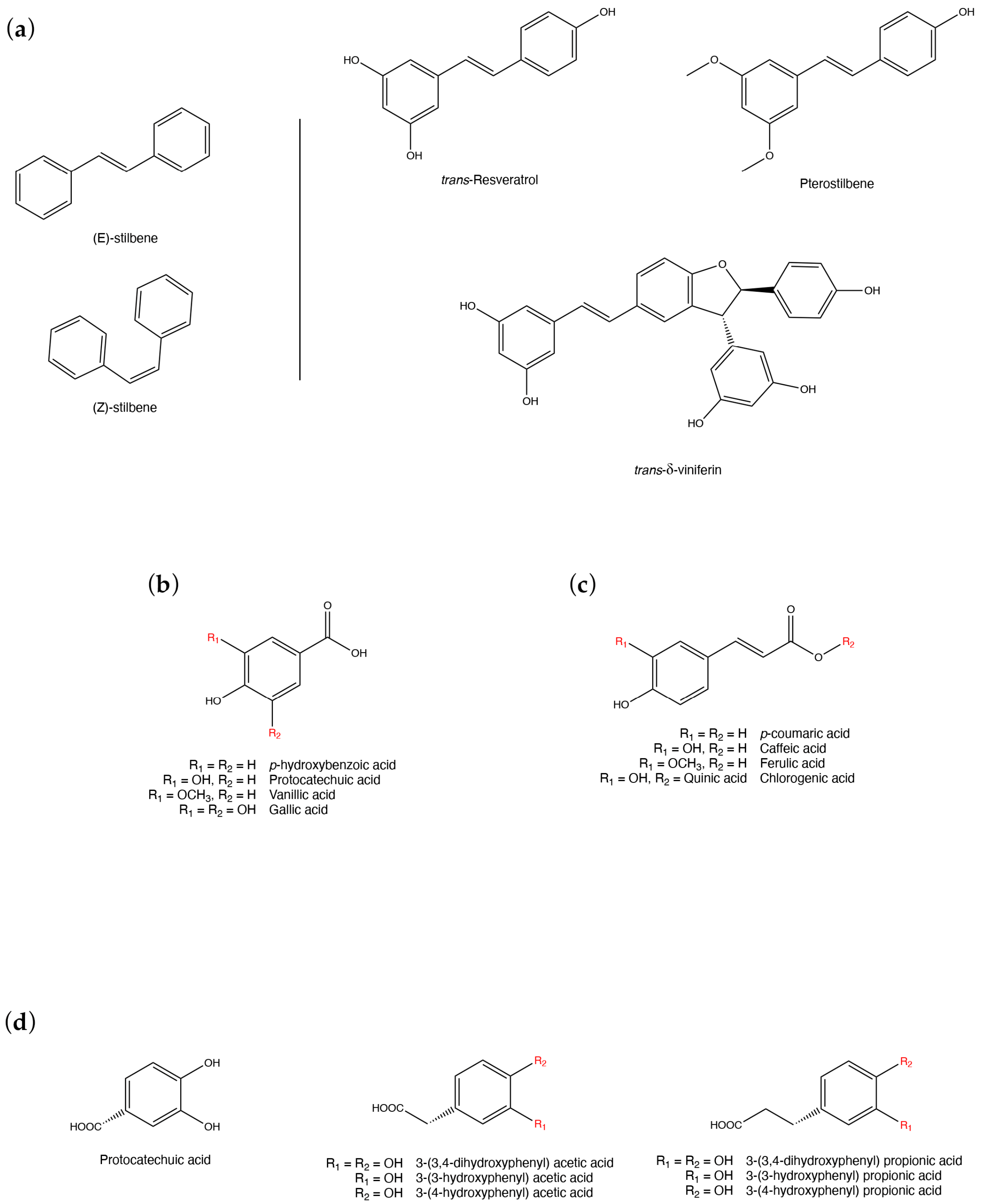

Supplementary Figure S2. Chemical structure of stilbenes, phenolic acids and gut microbiota-derived phenolic metabolites. (**a**) Chemical structure of stilbenes with example of the best known trans-stilbenoids (resveratrol, pterostilbene and trans-δ-viniferin). (**b**) Structure of hydroxybenzoic and (**c**) hydroxycinnamic phenolic acids. (**d**) Chemical structure of the main phenolic acids resulting from the gut microbial fermentation of phenolic compounds. Such compounds are absorbed in the portal circulation and are most likely responsible for the positive effects on the human body. When no substituent is specified in the "R" position, the presence of a hydrogen is implied.

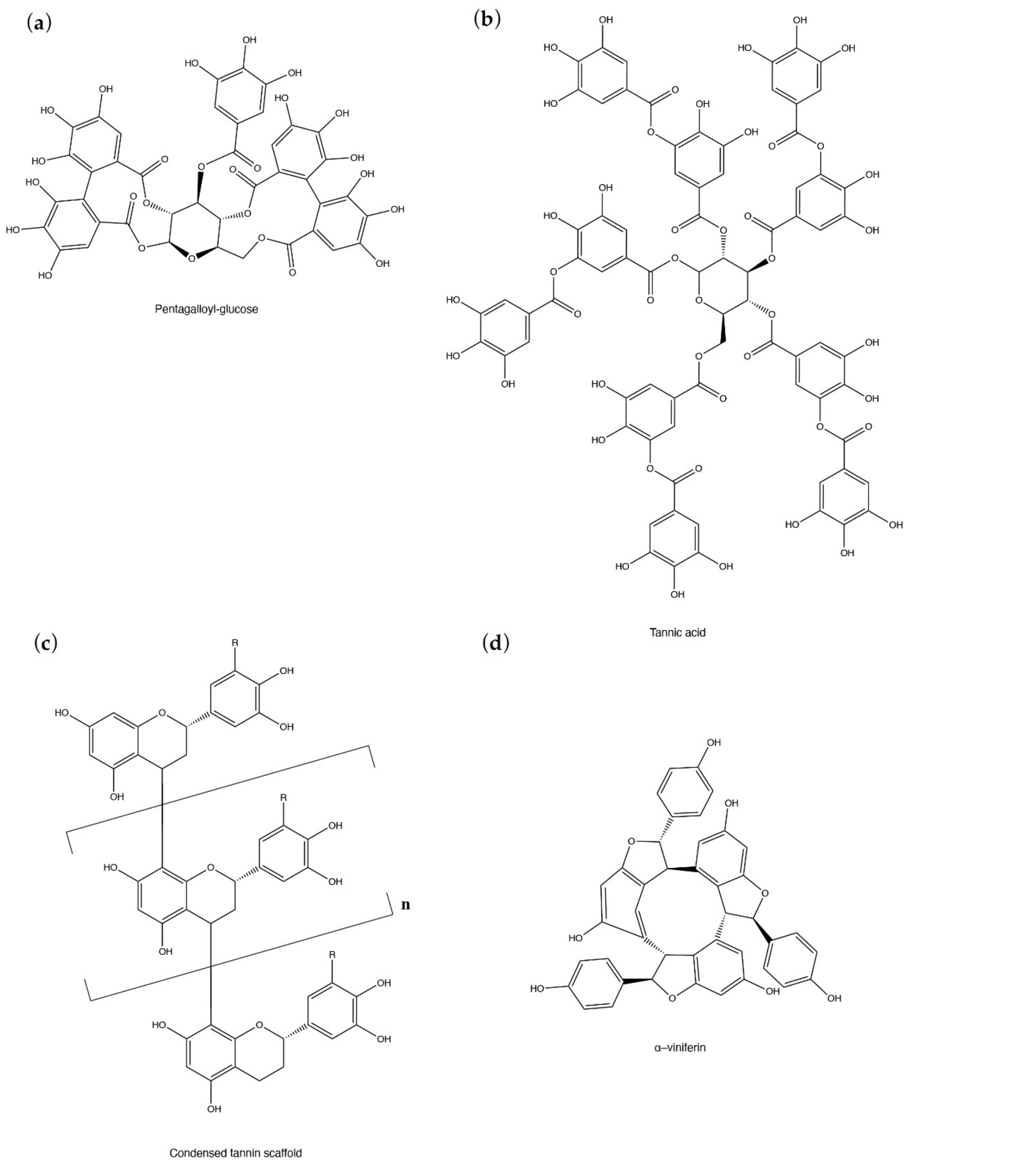

Supplementary Figure S3. Chemical structure of tannins. (**a**) Chemical structure of a hydrolysable tannin (pentagalloyl-glucose, made of five gallic acid units complexed to a glucose sugar core). (**b**) Tannic acid, another well-known example of hydrolysable tannin. (**c**) Example of condensed tannin structure. (**d**) High molecular weight viniferin - technically a polystilbene - but often considered a tannin due to its higher bulkiness compared to stilbenes or smaller viniferins.

Supplementary Table S1. Summarized information of cited *in vitro* and *in vivo* studies.

Phenolic class	Phenolic compound(s)	Administered dose	Model/study design	Administration duration	Ref
Flavonols	Quercetin	*In vitro*: 10, 20, 40, 80 and 120 µM<br>*In vivo*: 50-200 mg/kg	*In vitro*: CT-26, LNCaP, PC3, PC12, MCF-7, MOLT-4, U266B1 and CHO cell lines;<br>*In vivo*: female BALB/c mice, aged 6–8 weeks (weight, 20–25 g) bearing MCF-7 and CT-26 tumors	*In vitro test*: 48 h;<br>*In vivo test*: 40 days	13
	Epicatechin and quercetin	Randomized placebo-controlled study: daily consumption of 30 g enriched bread with 0.05% of a 1:1 mixture of (−)-epicatechin and quercetin<br>*In vitro*: 5–10 µM	In humans: 156 volunteers with risk factors for metabolic syndrome;<br>*In vitro*: Caco-2 cell line	Randomized placebo-controlled study: 3 months with weekly follow-up;<br>*In vitro* antioxidant testing on Caco-2 cells: 15–20 min;<br>*In vitro* DPPH assay: 60 min	14
	Flavonols	Estimated at 500 mg/day	In humans: >50,000 participants in the Danish Diet, Cancer and Health prospective cohort	23 years of dietary data collection (FFQ) and flavonoid content estimation	16
	Flavonols	15.6–1000 µg/mL	*In vitro: Staphylococcus aureus, Enterococcus faecalis, Escherichia coli, Pseudomonas aeruginosa*	*In vitro* MIC testing: 24 h	18
	Rutin	500 µM	*In vitro:* 10 human healthy donors	*In vitro* anaerobic fermentation: up to 24 h	24
	Rutin, naringin, quercetin, quercitrin, naringenin, hesperidin and hesperetin	0.1 mM	*In vitro:* 100 human healthy donors aged 20-72 years	*In vitro* α-L-rhamnosidase activity testing: 30 min	26
	Quercetin and related metabolites	*In vitro:* 10 nM - 1 mM<br>*In vivo:* 0.2-25 mg/kg	*In vivo*: 13 male normotensive Wistar rats obtained from MediTox (Czech Republic) and 6 SHR rats from Charles River (USA);<br>*In vitro*: isolated thoracic rat aortas	*In vitro:* 30 min for myorelaxation assay<br>*In vivo:* 5-min infusion or single bolus, then followed up to 120 min	28

Flavanones	*Citrus* flavanones	Flavanone mix used: FRAP: each flavanone (3.13–3200 µM); ORAC: each flavanone (0.08–800 µM); TEAC: each flavanone (2.0–64 µM); DPPH Assay: each flavanone (3.13–6400 µM); ADA: each flavanone (100–6400 µM); APA: each flavanone (6.25–140 µM)	*In vitro* chemical/enzymatic assays; *In vitro:* simulated human enzymatic and chemical digestion; *In vitro* anti-inflammatory assay on Caco-2 cell line	*In vitro* chemical/enzymatic assays: FRAP: 4 min; ORAC: 90 min; TEAC: 6 min; DPPH Assay: 20 min; ADA: 30 min; APA: 30 min; *In vitro* digestion: 2 h gastric phase, 4 h duodenal phase; *In vitro* anti-inflammatory activity testing: 24 h	31
	Citrus flavanones	250 µM	*In vitro*: Caco-2 cell line	*In vitro*: uptake experiment: 5 h; permeation experiment: 5 h	35
	Flavanones from *Macaranga Trichocarpa*	1.17–300 µg/mL	*In vitro*: *Bacillus subtilis*, *Enterobacter aerogenes*, *Escherichia coli*, *Pseudomonas aeruginosa*, *Salmonella* Typhi, *Shigella dysenteriae*, *Staphylococcus aureus* and *Vibrio cholerae*	*In vitro* MIC testing: 24 h	36
	Flavanones-rich extracts of *Calceolaria thyrsiflora* Graham	*In vitro* MIC testing: fungicidal: 50-250 mg/L; antibacterial: 12.5-1,600 µg/mL; *In vitro* cytotoxic activity: 0.01-100 µM	Fungal taxa: *Botrytis cinerea* and *Phytophtora cinnamomi*; Phytopathogens: *Agrobacterium tumefaciens* and *Bacillus subtilis*; Bacterial strains: methicillin-resistant *Staphylococcus aureus* (622-4 and 97-7), and *Escherichia coli* 33.1; *In vitro* cytotoxic activity: MDA-MB-231, B16-F10 and MEF cell lines	Fungicidal MIC: 120 h; Antibacterial MIC: 24 h; *In vitro* cytotoxic activity: 72 h	37
Isoflavones	Soy isoflavones	10 or 100 mg/mL	*In vitro* microtiter plate assays: pathogenic biofilms of *Listeria monocytogenes*, *Escherichia coli*, *Pseudomonas aeruginosa*, and methicillin-resistant *Staphylococcus aureus* (MRSA)	24 h	47
	Soy isoflavones	≥1,600 mg/kg	*In vivo:* 96 weaned barrows	Experimental isocaloric diets were fed over 7 feeding phases up to 161 days	49

	Genistein, daidzein and equol	0.1-100 µM	*In vitro:* HUVEC, HepG2 and Caco-2 cell lines	2 h	50
	Daidzein	1,200 µM	*In vitro* bacterial cultures: *Eggerthella* sp. Julong 732 and *Lactobacillus* sp. Niu-O16	Up to 72 h	52
	O-desmethylangolensin (O-DMA) and equol	estimated 83 mg daidzein from commercial soy bar and estimated 10 mg daidzein from package of soy nuts	In humans: 297 adults aged 18-95 years	3 days	55
Flavones	Synthetic flavone derivatives	*In vitro:* DPPH radical scavenging assay: 25-150 µg/mL; Hydrogen peroxide scavenging activity: 25–150 µg/mL; Lipoxygenase activity: 12.5-250 µg/mL; Anticholinesterase activity:12.5-250 µg/mL	*In vitro* chemical/enzymatic assays	*In vitro* chemical/enzymatic assays: DPPH assay: 30 min; Hydrogen peroxide scavenging activity: 10 min; Lipoxygenase activity: 1 min; Anticholinesterase activity: 15 min	56
	Flavone, apigenin and luteolin	*In vitro:* cell viability assay: 0–100 µM; Colony formation assay: 27-136 µM; Wound healing and cell migration assay: flavone (88 µM), apigenin (30 µM), and luteolin (43 µM)	*In vitro*: Hs578T, MDA-MB-231 and MCF-7 cell lines	*In vitro:* Cell viability assay: 72 h; Colony formation assay: 21 days; Wound healing assay: 24 h; Analysis of cell migration and invasion: monitored every hour for 9 h	58
	Flavone C-glucosides homoorientin and isovitexin	200 µM	*In vitro* bacterial cultures: *Eubacterium cellulosolvens* ATCC 4317	Up to 144 h	61
	Aronia berry extract	500 mg of extract	In humans: randomized placebo-controlled trial with 49 former healthy adult smokers	Up to 12 weeks	64
	Polymethoxyflavone	200 mg/kg	*In vivo:* 70 four-week-old male Institute of Cancer Research (ICR) mice	24 h	65
Flavan-3-ols	(+)-Catechin	*In vitro*: 5–25 µg/ml; *In vivo*: 200 µmol/kg	*In vitro:* HUVEC cell line; Sprague-Dawley rat aorta and dorsal aorta for ring assay; *In vivo*: 16 C57BL/6 mice	*In vitro:* up to 48 h on cells; *In vivo:* 9 days	66

	Catechin and procyanidin B2 co-administered with DOCE	*In vitro*: Catechin: 5-100 µM; ProB2 0.1-50 µM; DOCE 1-120 nM	*In vitro*: MCF-7, T47D, MDA-MB-231, PC3 and DU145 cell lines	*In vitro:* coincubation up to 72 h; clonogenic assays: 14 days	67
	Catechin hydrate	2-2048 µg/mL	*In vitro:* 20 *Staphylococcus aureus* strains isolated from clinical wound samples, and three reference strains of *S. aureus*: ATCC 25923, *S. aureus* ATCC 43300 and *S. aureus* ATCC 6538	*In vitro* MIC testing: 24 h	75
	Mango (*Mangifera indica* L.) extract	estimated 190.36 mg in 400 g of mango pulp; participants were asked to include 200 to 400 g of mango pulp every day	In humans: 14 volunteers with diagnosed IBD, aged 18-75 years	8 weeks	76
	Theaflavin-3,3′-digallate (TFDG); epigallocatechin gallate (EGCG)	*In vitro* fermentation: TFDG 50 µM; EGCG 100 µM	*In vitro* fermentation with 4 healthy donor slurries (age, 24–38 years)	48 h	79
	5-(3′,4′-dihydroxyphenyl)-γ-valerolactone and sulphate conjugates	1000 µM	*In vitro*: T24 bladder epithelial cell lines (ATCC® HTB4™); UPEC strain (ATCC®53503™) for adhesion assays	*In vitro:* adherence assays: 1 h; cytotoxicity assay: 24 h	81
Anthocyanins	Anthocyanins from *Nitraria tangutorun* Bobr. fruits	*In vitro:* 0-4 mg/mL for DPPH and radical scavenging assays; *In vivo:* 0.35-2.10 g/kg	*In vivo*: 72 female and male Wistar rats (160 ± 15 g)	*In vitro*: 30 min for radical-scavenging assay; *In vivo*: 4 weeks	83
	Anthocyanin-enriched purple corn extract	Estimated 0.24 ± 0.01 mg of anthocyanins/g of food pellets; Food consumption of 22.5 ± 0.6 g/J (*i.e.*, ~64 kcal)	*In vitro:* 60 male Wistar rats (75-100 g)	8 weeks	85
	Anthocyanin-enriched purple corn extract	53 mg of anthocyanins/kg	*In vivo:* 70 male adult Sprague-Dawley rats (200–250 g)	72 h	86
	Anthocyanins-rich extract from wild Norwegian wild berries (*Vaccinium myrtillus*) and blackcurrants (*Ribes nigrum*)	320 mg anthocyanins/day (two 80-mg capsules in the morning and two 80-mg capsules in the afternoon, each day)	In humans: 16 healthy volunteers with BMI<25 kg/m^2 (normal weight)	28-day administration, followed by a 2-week wash-out period, then cross-over supplementation	88

	Anthocyanins-rich extract from wild Norwegian wild berries (*Vaccinium myrtillus*) and blackcurrants (*Ribes nigrum*)	320 mg anthocyanins/day (two 80mg capsule in the morning and two 80mg capsule in the afternoon, each day)	In humans: 26 volunteers with BMI>25 kg/m^2 (obese and overweight)	28-day administration, followed by a 2-week wash-out period, then cross-over supplementation	89
	Phloroglucinol (1,3,5-benzenetriol; Flospan®)	160 mg administered orally 3 times a day for 2 weeks.	In humans: 72 patients with IBS-D	2 or 3 weeks	95
	Phloroglucinol (1,3,5-benzenetriol; Flospan®)	Oral phloroglucinol 160 mg, single dose	In humans: 134 EGD outpatients aged 18–80 years	Administered single dose 15 min before EGD	96
	Anthocyanins-rich extract from *Ipomoea batatas* L.	*In vitro* estimated concentration of anthocyanins used: 0.5-2.5 mg/mL for DPPH assay; MIC assay 25-3000 µg/mL; Anti-proliferative activity: 1-5 mg/mL	*In vitro*: *Bifidobacterium infantis, Bifidobacterium adolescentis, Bifidobacterium bifidum, Lactobacillus acidophilus, Staphylococcus aureus* and *Salmonella typhimurium*	DPPH assay: 30 min; Superoxide anion scavenging capacity analysis: 4 min; Total reducing power (TRP) analysis: 20 min; Ferrous ion-chelating activity analysis: 10 min; Effects on probiotic bacteria proliferation: 36 h; MIC: 12 h; Anti-proliferative activity on *S. aureus* and *S. typhimurium*: 12 h	98
Stilbenes	Stilbenes-rich extract from grapevine shoot (ST-99 extract)	0.1-1000 µg/ml	*In vitro*: Caco-2 cell (ATCC® HTB-37), HepG2 cells (ATCC® HB-8065) cell lines	*In vitro* cytotoxicity and oxidative stress assays: 48 h	107
	ε-Viniferin, trans-resveratrol, ST-99 extract	90-360 mg ST-99/kg	*In vivo*: 56 nine-week-old Wistar rats, strain RjHan: WI (type outbred rats)	45 h	108
	Vitis vinifera extract from grapevine shoots (around 30% stilbene content)	0-4 g/L of extract	*In vitro:* 1 healthy human female donor aged 30 years	Up to 13 days in the M-SHIME® *in vitro* fermentation system	111
	trans-resveratrol, oxyresveratrol, batatasin III, piceatannol, thunalbene, and pinostilbene	15 µg/mL	5 healthy human donors aged 23-29 years, with BMI of 24.6 kg/m^2	*In vitro* simulation of colonic fermentation with donor slurry for up to 48 h	112

trans-resveratrol	*In vitro* incubation with bacterial species: 23 mM; fermentation with donor slurry: 32-mM solution of trans-resveratrol; *In vivo:* 0.5 mg/kg in dietary intervention single dose	*In vitro*: bacterial cultures of *Adlercreutzia equolifaciens, Bifidobacterium pseudocatenulatum, Eggerthella lenta, Faecalibacterium prausnitzii, Slackia equolifaciens, Clostridium scindens, Slackia isoflavoniconvertens, Eubacterium rectale, Bacteroides plebeius, Lactococcus garvieae, Peptoniphilus harei, Finegoldia magna, Bifidobacterium animalis, Bifidobacterium infantis, Bifidobacterium longum, Bifidobacterium bifidum*, and *Bifidobacterium* spp. MRI-F 45, 48, and 49, *Bacteroides ovatus, Bacteroides vulgatus, Citrobacter murliniae, Escherichia coli, Enterococcus faecium*, and *Lactobacillus salivarius*; *In vitro* fermentation of donor slurry: 7 healthy human donors aged 26-54 years; In humans: 12 healthy volunteers aged 18-28 years, BMI 20-26 kg/m^2 for dietary intervention	*In vitro:* up to 48 h for fermentation with bacterial species or donor slurry; In humans: 24-h follow-up for dietary intervention after single dose	113
trans-resveratrol	500 mg oral supplementation, once a day	In humans: 60 healthy subjects aged 18-29 years	28 days	115
trans-resveratrol	250 or 500 mg per dose	In humans: 24 healthy subjects aged 18-29 years	7 days for bioavailability and cognitive assessments	116
Ramizol	0.008-8 µg/mL	*In vitro:* 100 *Clostridioides difficile* clinical isolates selected from the Micromyx collection, as well as the American Type Culture Collection (ATCC) quality control organism *Clostridioides difficile* ATCC 700057	48 h for antimicrobial susceptibility testing	118
Ramizol	0, 50, 500, and 1500 mg/kg/day	*In vivo:* CRL Sprague-Dawley CD® IGS rats (Charles River Laboratories, Inc.) 8-week-old, 233-263 g (males) and 183-226 g (females) with *Clostridioides difficile* colitis infection	24 h for toxicokinetic; 15 days for overall treatment	119

	ferulic acid	1, 5, 10, 50, 100, 200, 300, and 500 µg/mL	*In vitro:* NIH-3T3, 3T3-L1, HepG2 cell line; *In vivo:* male imprinting control region (ICR) mice (20–30 g), 6-week-old	48 h on cells; 10 min for hematological fractions from mice and for human platelet-poor plasma; 5 min for platelet function assay with PFA-100 instrument; 5 min for ATP and serotonin release assays with thrombin	120
Phenolic acids	*p*-coumaric acid	25–500 µg/mL	*In vitro:* PC12 cell line	30 min for DPPH radical scavenging assay; 12-16 h for $ABTS^{•+}$ radical scavenging assay; 10 min for ferric reducing power assay; 15 min for metal-ion chelating activity; 2 h on PC12 cell line; 2 h for LHD release rate assay; 2 h for ROS production, MDA, SOD and CAT activity; *in vivo* for 6 weeks	121
	Gallic acid, methyl gallate	0.003, 0.01 and 0.03 mg/mL	Calcium oxalate precipitation *in vitro* assay	60 min for calcium oxalate precipitation	122
	Vanillic acid	*In vitro:* 0, 3, 10, 30 µM; *In vivo:* 10 and 30 mg/kg	*In vitro:* HCT116, Hep3B, A549, and HUVEC cells; *In vivo:* male BALB/c nude mice (4–5 weeks of age, 20 ± 2 g)	*In vitro:* 12 h for HIF-1a luciferase reporter, MTT, tube formation, chlorogenic assays and flow cytometry analysis; *In vivo:* 50 days for *in vivo* xenografted tumor model	123
	Phenolic acids and modified phenolic acids used as analogs after incorporation in polyurethane biomaterial	0.078-5 mg/mL	*In vitro:* NIH/3T3 Swiss mouse fibroblasts (ATCC–CCL92); *Escherichia coli* (397E strain), *Staphylococcus epidermidis* (native (FDA strain PCI 1200) and drug-resistant (4483 strain: multi-drug resistant)) and *Staphylococcus aureus* (native (Wichita strain) and drug-resistant (F-182 strain: methicillin and oxacillin resistant))	3 h, 24 h for cytocompatibility assay; 3 h for protective effects against hydrogen peroxide toxicity test; 24 h for antimicrobial IC50 testing; 4 h, 24 h for CFU inhibition quantification	124
	Chlorogenic acid, caffeic acid	Serial dilution of the starting solution at 60 mg/mL for chlorogenic acid and 50 mg/mL for caffeic acid	*In vitro*: *Listeria monocytogenes* EGDe strain	24 h for MIC assay and bacterial cell growth study	125

	Chlorogenic acid	*In vitro:* 8-4096 µg/mL;<br>*In vivo:* 500 mg/kg	*In vitro:* KPN 17489 (biofilm negative) and KPN 19936 (biofilm positive);<br>*In vivo:* male C57BL/6 mice (8 weeks of age) with body weight 22 ± 2 g	*In vitro:* 24 h for MIC assay; 5 days for biofilm inhibition, with fresh medium every 24 h;<br>*In vivo:* 48 h for *in vivo* testing of KPN biofilm inhibition	126
	Dry-blanched peanuts and peanuts skins; protocatechuic, p-coumaric, and ferulic acids	100 ppm equivalents from food extract; 500, 250, 100, 50, 10 and 5 mg/mL	In vitro: *Bacillus cereus* (IAL 55), *Staphylococcus aureus* (ATCC 13565), *Geobacillus stearothermophilus* (ATCC 7953), *Listeria monocytogenes* (ATCC 7644) and Gram-negative bacteria *Pseudomonas fluorescens* (ATCC 13525), *Pseudomonas aeruginosas* (IAL 1853), *Escherichia coli* (IAL 2064), *Salmonella enteritidis* (S 2887), and *Salmonella* Typhi (IAL 2431)	24 h for MIC assay; 14 days for antioxidant activity in gamma-irradiated fish model system	127
	Coffee (with quantified amount of chlorogenic acids)	412 µmol single dose administered	In humans: 11 healthy participants aged 19-35 years, average BMI 24.3 ± 2.3 kg/m^2	Single dose administered, followed by urine monitoring for 24 h	128
	Caffeic acid	1.52 mg dose, equivalent to ∼6.08 mg/kg body weight	*In vivo:* Male Sprague–Dawley rats ∼250 g	Single dose administered, followed by monitoring for 72 h	129
	Plant extract rich in tannins, flavonoids and phenolic acids	10 mg/mL stock extracts, tested at different dilutions	*In vitro* chemical assays	30 min for DPPH radical scavenging assay; 10 min for reducing power assay; 180 min for β-carotene bleaching system	131
Tannins	White clover cultivar “Grasslands Mainstay” extract, rich in tannins	500mg for *in vitro* fermentation	Rumen fluid from fistulated pasture-fed cows	24 h for rumen fluid *in vitro* fermentation	132
	Anthemis praecox Link (locally named “El baboon”) extract rich in tannins, flavonoids, and phenolic acids	100–1000 µg/mL for DPPH; 0.1 and 1 mg/mL for ABTS; 1, 5 and 10 mg of the dried extract for antibacterial activity	*In vitro:* Gram-positive bacteria (*Staphylococcus aureus* ATCC 25923, *Enterococcus faecalis* ATCC 29212), and Gram-negative bacteria (*Escherichia coli* ATCC 25922, *Pseudomonas aeruginosa* ATCC 27853, *Salmonella* Typhi ATCC 14028, *Klebsiella pneumoniae* ATCC 700603)	30 min for DPPH radical scavenging assay; 12-16 h for $ABTS^{•+}$ radical scavenging assay; 2 h for β-carotene bleaching assay; 24 h for antibacterial activity	136

Plant extract rich in tannins	*In vitro* fermentation with donor slurry: food prepared with 0.6% w/w of different tannin extracts with 72-92% tannin content	*In vitro* digestion of prepared food, first enzymatically pre-digested in an upper digestive tract model, then fermented with six human volunteer stools with BMI 18.5-25 kg/m^2	2 min for alpha amylase simulated oral digestion; 2 h for simulated gastric fluid digestion; 2 h for simulated bile acid and pancreatic fluid intestinal digestion; 20 h for fermentation with human slurry	142

ABTS, 2,2′-azinobis-(3-ethylbenzothiazoline-6-sulfonic acid) radical; ADA, Anti-Drug Antibody; APA, Anti-Proliferative Antibody; BALB/c mice, Albino immunodeficient mice; BMI, Body Mass Index; CAT, Catalase; CFU, Colony-Forming Unit; DOCE, Docetaxel; DPPH, 2,2-Diphenyl-1-picrylhydrazyl; EGD, Esophagogastroduodenoscopy; FFQ, Food Frequency Questionnaire; FRAP, Ferric Reducing Ability of Plasma; HIF, Hypoxia-Inducible Factor; IBD, Inflammatory Bowel Disease; IBS, Irritable Bowel Syndrome; IBS-D, Diarrhea phenotype of IBS; IC50, concentration responsible for the reduction of bacterial CFU by 50%; KPN, *Klebsiella pneumoniae*; MDA, Malonic Aldehyde; MIC, Minimum Inhibitory Concentration; MRSA, Methicillin-resistant *Staphylococcus aureus*; MTT, 3-[4,5-dimethylthiazol-2-yl]-2,5 diphenyl tetrazolium bromide; O-DMA, O-Desmethylangolensin; ORAC, Oxygen Radical Absorbance Capacity; ProB2, Procyanidin B2; ROS, Radical Oxygen Species; SHR rat, Spontaneously hypertensive rat; SOD, Superoxide Dismutase; TEAC, Trolox Equivalent Antioxidant Capacity; TRP, Total Reducing Power.

Cell lines: 3T3-L1, Mouse fibroblast cell line from Swiss albino mouse embryo tissue; A549, Human hypotriploid alveolar basal epithelial cells derived from pulmonary carcinoma tissue; Caco-2, Human originally derived from a colon carcinoma; CHO, Epithelial cell line derived from the ovary of the Chinese hamster; CT-26, N-nitroso-N-methylurethane-(NNMU) induced, undifferentiated human colon carcinoma; DU145, Human cerebral metastasis of prostate cancer; HCT116, Human colon cancer cell line; Hep3B, Human liver cancer cell line; HepG2, Human cell line derived from well-differentiated hepatocellular carcinoma; Hs578T, Human epithelial cell line isolated from breast tissue carcinoma; HUVEC, Human endothelial cell line that was isolated from the vein of the umbilical cord;

LNCaP, Androgen-sensitive human prostate adenocarcinoma; M16-F10, Murine melanoma cell line; MCF-7, Human epithelial cell line isolated from the breast tissue of a patient with metastatic adenocarcinoma; MDA-MB-231, Epithelial, human breast cancer cell line that was established from a pleural effusion of a 51-year-old Caucasian female with a metastatic mammary adenocarcinoma; MEF, Mouse embryonic fibroblasts; MOLT-4, T lymphoblast human cell line from acute lymphoblastic leukemia patient in relapse; NIH-3T3, Fibroblast cell line that was isolated from a mouse NIH/Swiss embryo; PC12, Transplantable rat pheochromocytoma; PC3, Human bone metastasis of a grade IV prostatic adenocarcinoma; T47D, Human epithelial cells isolated from infiltrating ductal breast carcinoma; U266B1, B lymphocyte cell line isolated from the peripheral blood of a 53-year-old, male patient with myeloma.
